# Gradient Patterns of Age-Related Diffusivity Changes in Cerebral White Matter

**DOI:** 10.3389/fneur.2022.870909

**Published:** 2022-06-02

**Authors:** Jasmina Boban, Majda M. Thurnher, Nikola Boban, Meng Law, Neda Jahanshad, Talia M. Nir, Dajana F. Lendak, Dusko Kozic

**Affiliations:** ^1^Faculty of Medicine Novi Sad, Department of Radiology, University of Novi Sad, Novi Sad, Serbia; ^2^Vojvodina Institute of Oncology, Center for Diagnostic Imaging, Sremska Kamenica, Serbia; ^3^Department for Biomedical Imaging and Image-guided Therapy, Medical University Vienna, Vienna, Austria; ^4^Clinical Center of Vojvodina, Center for Radiology, Novi Sad, Serbia; ^5^Department for Neuroscience, The Alfred Centre, Central Clinical School, Monash University, Melbourne, VIC, United States; ^6^Imaging Genetics Center, Mark and Mary Stevens Neuroimaging and Informatics Institute, Keck School of Medicine, University of Southern California, Marina del Rey, CA, United States; ^7^Faculty of Medicine Novi Sad, Department of Infectious Diseases, University of Novi Sad, Novi Sad, Serbia; ^8^Clinical Center of Vojvodina, Clinic for Infectious Diseases, Novi Sad, Serbia

**Keywords:** diffusion-tensor imaging, brain aging, magnetic resonance imaging, physiological brain aging, pattern

## Abstract

The current concept of brain aging proposes three gradient patterns of changes in white matter that occur during healthy brain aging: antero-posterior, supero-inferior, and the myelodegeneration-retrogenesis (or the “last-in-first-out”) concept. The aim of this study was to correlate white matter diffusivity measures (fractional anisotropy-FA, mean diffusivity-MD, radial diffusivity-RD, and axial diffusivity-AD) in healthy volunteers with chronological age and education level, in order to potentially incorporate the findings with proposed patterns of physiological brain aging. The study was performed on 75 healthy participants of both sexes, with an average age of 37.32 ± 11.91 years underwent brain magnetic resonance imaging (MRI) with diffusion tensor imaging (DTI). DTI was performed using tract-based spatial statistics (TBSS), with the analysis of four parameters: FA, MD, RD, and AD. Skeletonized measures were averaged in 29 regions of interest in white matter. Correlations between age and DTI measures and between education-level and DTI measures were performed using Pearson's correlation test. To correct for multiple comparisons, we applied a Bonferroni correction to the *p*-values. Significance was set at *p* ≤ **0.001**. A significant negative correlation of FA with age was observed in posterior thalamic radiation (PTR) (*p*< **0.001**). A significant positive correlation between age and MD was observed in sagittal stratum (SS) (*p*< **0.001**), between age and RD in PTR, SS, and retrolenticular internal capsule (*p*< **0.001**), and between age and AD in the body of the corpus callosum (*p*< **0.001**). There were no significant correlations of DTI parameters with educational level. According to our study, RD showed the richest correlations with age, out of all DTI metrics. FA, MD, and RD showed significant changes in the diffusivity of projection fibers, while AD presented diffusivity changes in the commissural fibers. The observed heterogeneity in diffusivity changes across the brain cannot be explained by a single aging gradient pattern, since it seems that different patterns of degradation are true for different fiber tracts that no currently available theory can globally explain age-related changes in the brain. Additional factors, such as the effect of somatosensory decline, should be included as one of the important covariables to the existing patterns.

## Introduction

Healthy brain aging occurs as a result of numerous interconnected structural, chemical, and functional brain changes, and, in turn, can lead to a decline in cognitive function. Brain aging is associated with a decline in concentration, attention, and other executive functions, as well as global cognitive information processing (1). Optimal cognitive functioning, which is vital for independent living, productivity, and overall quality of life, relies on coordinated processes in different brain regions. Disturbances in communication (i.e., disconnections) between these regions during healthy aging may result in cognitive decline (2).

Over the years, many novel techniques of magnetic resonance imaging (MRI) were introduced in order to clarify the neuropathological process that lies behind neurological and psychiatric disorders. One of the most revolutionary techniques is diffusion-weighted imaging (DWI) which provides tissue contrast based on the magnitude of diffusion differences between water molecules (3). Diffusion of the water molecules represents the random motion of the molecules (Brownian motion), and it is restricted or facilitated depending on the ongoing pathological process (4). The more advanced technique, derived from DWI is called diffusion-tensor imaging (DTI) that analyzes the three-dimensional shape of diffusion, named diffusion tensor. The diffusion tensor is actually a 3D structure defined by three principal diffusivities (eigenvalues, λ1, λ2, and λ3) that are associated with three principal directions (eigenvectors) (5). This technique requires more robust post-processing but can provide valuable information about the microstructure of the brain tissue.

The concept of cortical disconnections has been confirmed in healthy brain aging (without the manifest neurological disorder), using this technique in previous studies (6–8). Given that DTI is a non-invasive imaging method, it represents a useful tool to probe brain network integrity and functionality (9). DTI fractional anisotropy (FA), mean diffusivity (MD), radial diffusivity (RD), and axial diffusivity (AD) measures can give insight into diffusivity changes in the brain which can be driven both by physiological brain aging and pathological processes (degeneration, inflammation, neoplastic and other diseases). Most published DTI studies on the aging brain have relied on the FA, which has been shown to decrease throughout the white matter of the brain during the aging process (10–12). Age-related increases in MD and RD have also been consistently observed (13). However, findings regarding AD have been inconsistent, with both increases and decreases observed in different settings (10, 13). The studies comprehensively exploring all four DTI metrics mainly explored pathological processes in the brain (neurodegenerative and genetic disorders) (14, 15). There are several methods for the evaluation of DTI data, with tract-based spatial statistics (TBSS) being one of the most popular and also used in this study. The main idea of the TBSS approach is to project volumetric data onto a white matter skeleton, in order to gain statistical power and skip some steps regarding data processing (16).

There are three proposed gradient patterns of white matter changes during healthy brain aging. The first is the antero-posterior pattern, with more extensive changes in the anterior/frontal parts of the brain preceding the posterior lobes (17). The second pattern is the supero-inferior, with extensive changes present in the cranial aspects of the brain parenchyma (18). The third pattern is myelodegeneration-retrogenesis or, the “last-in-first-out” hypothesis, which proposes that the degeneration of myelin observed during the aging process occurs in the opposite direction of myelin development and maturation (19, 20).

The aim of this study was to correlate white matter DTI anisotropy and diffusivity measures (FA, MD, RD, and AD) using TBSS in healthy volunteers, with chronological age and formal education level (expressed in years of education). The second aim was to determine whether the gradient pattern of white matter associations was consistent with any proposed patterns of physiological brain aging.

## Materials and Methods

### Study Population

Of a total of 81 healthy volunteers who were enrolled in the study based on the initially performed power sample analysis (α = 0.05 (*p*-value), β = 0.2 (correspondent to the ower of 80%), and *r* > 0.30, the required sample was 79), 75 participants, average age 37.32 ± 11.91 years (range: 22–62 years; 53 male and 22 female participants) were included into analyses after undergoing whole brain magnetic resonance imaging (MRI) from July 2011 to April 2017 at the University of Novi Sad. Six data sets were excluded from the initial sample of 81 subjects due to technically inadequate quality. All the patients were cognitively screened using Mini-Mental State Examination (MMSE) (21).

Inclusion criteria were over 18 years of age, MMSE score over 24 (thus excluding persons eligible for the evaluation of dementia), and right-handed. MMSE is a 30-point screening test for the global cognitive assessment, used for quick exclusion of subjects with signs of cognitive impairment. Right-handedness was based on the self-report of the preferred hand and on the results of the Waterloo Handedness Questionnaire (WHQ) (22). Criteria for the exclusion from the study were acute and chronic neurologic and psychiatric disorders, presence of diffuse or focal white matter lesions in the brain (tumors, infarctions, metastases, vascular malformations, white matter hyperintensities), post-operative state, head trauma history, patients with palsy or deep paresis of the dominant hand, visual and hearing disorders, MMSE score ≤ 24, history of drug and alcohol abuse according to Drug Abuse Screening Test and Michigan Alcohol Screening Test (MAST) (23), and contraindications for MRI scanning.

### Neuroimaging

All participants underwent an MRI of the brain on a 3T clinical scanner (Siemens Trio Tim, Erlangen, Germany), using an 8-channel head array. Conventional MRI of the brain consisted of T1W sagittal spin echo [time of repetition (TR)/time of echo (TE) 440 ms/3.8 ms, slice thickness 5 mm, duration 2:00 min], T2W transversal turbo spin echo (TR/TE 5150 ms/105 ms, slice thickness 5 mm, duration 2:57 min), Fluid Attenuation Inversion Recovery (FLAIR) transversal (TR/TE 8,000 ms/101 ms, slice thickness 5 mm, duration 3:30 min), diffusion-weighted imaging (DWI) (TR/TE 4100 ms/91 ms, slice thickness 5 mm, duration 2:07 min), T2W coronal turbo spin echo (TR/TE 7150 ms/111 ms, slice thickness 5 mm, duration 2:17 min), and 3D T1W MPRAGE sagittal tomograms (TR/TE 1530 ms/2.97 ms, slice thickness 1 mm, duration 5:12 min).

Conventional MRI was necessary for obtaining anatomic information and detection of potential focal or diffuse brain lesions.

Diffusion tensor imaging was performed using MDDWI sequence (multidirectional diffusion weighted imaging) with two diffusion shells (b-values of 1,000 s/mm^2^ and 1,500 s/mm^2^) from 64 diffusion-weighted directions each and two non-diffusion weighted volumes (at b0). The data were acquired at 2 mm isotropic resolution; the fold over direction was A-P with a P shift. Field-of-view was 230. DWI images were denoised using the LPCA filter and corrected for motion by linearly aligning all DWI volumes to the b 0 image. T1-weighted images were denoised using the non-local means filter and underwent N3 intensity inhomogeneity normalization, and brain extraction. Data sets that did not fulfill the quality control criteria were excluded from the analyses (three due to extreme EPI distortion and three due to skeleton misregistration). T1 images were linearly aligned to diffusion images, and diffusion images were then non-linearly warped to their respective T1-weighted scans to correct for echo-planar imaging (EPI) induced susceptibility artifacts.

Diffusion gradient directions were rotated to accommodate linear registrations. DTI fractional anisotropy (FA), mean diffusivity (MD), radial diffusivity (RD), and axial diffusivity (AD) scalar maps were generated from corrected images (24).

Diffusion tensor imaging analysis was performed using the tract-based spatial statistics (TBSS) technique from the FSL software package (24) and publicly available ENIGMA-DTI protocols (http://enigma.usc.edu/protocols/dti-protocols/) (25). Each subject's FA map was warped to the ENIGMA-DTI FA template with ANTs (26) and the transformations were applied to all respective DTI maps. DTI measures were then projected onto the template skeleton. Skeletonized measures were averaged in a total of 29 regions of interest (ROIs) from the John Hopkins University White Matter Atlas (27) (Table 1).

**Table 1:** List of analyzed locations (ROIs) according to the John Hopkins White Matter Atlas.

**Location**	**Abbreviation**
Anterior corona radiate	ACR
Anterior limb of internal capsule	AIC
Body of the corpus callosum	BCC
Corpus callosum	CC
Cingulate gyrus	CGC
Parahippocampal cingulate fibers	CGH
Corticospinal tract	CST
External capsule	EC
Fornix	FX
Fornix-stria terminalis	FXST
Genu of the corpus callosum	GCC
Inferior fronto-occipital fasciculus	IFO
Posterior corona radiata	PCR
Posterior thalamic radiation	PTR
Posterior limb of the internal capsule	PLIC
Retrolenticular limb of internal capsule	RLIC
Sagittal stratum	SS
Splenium of the corpus callosum	SCC
Superior fronto-occipital fasciculus	SFO
Superior longitudinal fasciculus	SLF
Uncinate fasciculus	UNC

### Statistical Analysis

Statistical analysis was performed using the software package SPSS ver. 23.0 (IBM, Chicago, USA). Methods of descriptive and comparative statistics were used (mean, median, standard deviation, minimum, maximum, frequencies, and percentage, depending on the type of the variable). After confirmation of normal distribution, correlations between age and DTI measures and between education-level and DTI measures were performed using Pearson's correlation test. Additionally, a partial correlation was performed between age and diffusivity changes in observed DTI parameters, with education as a control variable. Permutation tests for Pearson's correlations were performed for age and localization, education and localization, and for partial correlation, using education as a control variable. Gender differences were also explored using the *t*-test.

To correct for multiple comparisons, we applied a Bonferroni correction to the *p*-values. Significance was set at *p* ≤ 0.001 (21 ROIs × 2 tests + 4 = 46, 0.05/42 = 0.00108).

## Results

A total of 75 participants were included in the study, 53 men (70.7%) and 22 women (29.3%). No significant gender-related differences in white matter diffusivity metrics were observed. The average age of the participants was 37.32 ± 11.91 years (range 22–62). The average education level was 13.87 ± 2.38 years of formal education. MMSE scoring was 28.87 ± 1.14 points, no person scored lower than 26.

**Figure 1:**
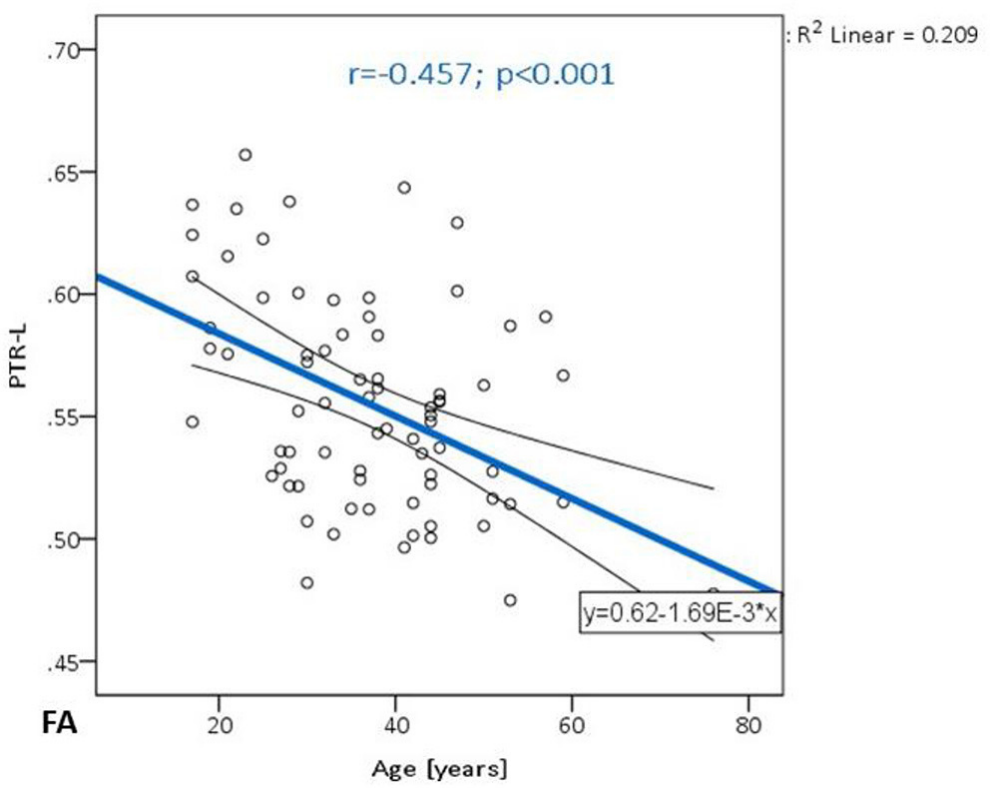
The trend of FA decrease with advancing age in the posterior thalamic radiation on the left.

Table 2 summarizes the changes in FA on the observed localization in the brain, in correlation with the chronological age and educational level of the participants. A significant negative correlation was observed in PTR (on the left *p*< **0.001** and on the right *p* = **0.001**) (Figure 1). All correlations were negative. No significant correlations of FA with the level of education or the MMSE score were detected. Table 2A shows the results of the partial correlation summarized.

**Table 2:** Results of Pearson's correlation of FA with age and education level of the study participants in the observed locations (*r*-correlation coefficient).

**Location**	**Age**		**Education level**	
	***r***	***p***	***r***	***p***
ACR-L	−0.258	**0.026**	**0.024**	0.841
ACR-R	−0.244	**0.035**	0.050	0.669
ALIC-L	**0.023**	0.843	−0.157	0.179
ALIC-R	0.060	0.608	−0.129	0.269
BCC	0.116	0.324	−0.197	0.090
CC	**0.040**	0.736	−0.215	0.064
CGC-L	**−0.038**	0.749	**−0.046**	0.693
CGC-R	0.079	0.499	−0.054	0.644
CGH-L	−0.114	0.329	0.055	0.642
CGH-R	−0.128	0.272	0.160	0.171
CR-L	−0.294	**0.010**	**−0.001**	0.992
CR-R	−0.293	**0.011**	**−0.012**	0.922
CST-L	**−0.002**	0.986	**0.014**	0.908
CST-R	**−0.004**	0.971	0.091	0.437
EC-L	−0.102	0.382	**0.038**	0.747
EC-R	−0.102	0.386	0.074	0.530
FX	−0.235	**0.043**	0.185	0.112
FXST-L	–**0.049**	0.679	0.100	0.395
FXST-R	−0.111	0.345	0.153	0.190
GCC	−0.159	0.172	−0.119	0.311
IC-L	−0.197	0.091	−0.087	0.458
IC-R	−0.131	0.264	−0.080	0.496
IFO-L	**0.002**	0.989	−0.188	0.107
IFO-R	**0.002**	0.989	−0.057	0.624
PCR-L	−0.226	0.052	−0.144	0.217
PCR-R	−0.229	**0.048**	−0.233	**0.044**
PLIC-L	−0.113	0.335	−0.145	0.216
PLIC-R	−0.121	0.301	−0.162	0.165
PTR-L	**−0.457**	**<0.001**	−0.056	0.634
PTR-R	**−0.366**	**0.001**	**0.020**	0.864
RLIC-L	**−0.351**	**0.002**	0.082	0.482
RLIC-R	−0.247	**0.033**	0.100	0.394
SCC	0.108	0.358	−0.230	**0.047**
SCR-L	−0.244	**0.035**	**0.031**	0.792
SCR-R	−0.248	**0.032**	**0.018**	0.875
SFO-L	**0.001**	0.992	−0.085	0.466
SFO-R	−0.098	0.401	0.098	0.401
SLF-L	−0.189	0.105	**−0.022**	0.853
SLF-R	−0.155	0.183	**−0.034**	0.770
SS-L	**−0.341**	**0.003**	0.121	0.300
SS-R	**−0.340**	**0.003**	0.161	0.167
UNC-L	−0.297	**0.010**	−0.280	**0.015**
UNC-R	−0.100	0.392	−0.318	**0.005**

*Significant results are presented in bold case*.

**Table 2A:** The results of partial correlation of FA with age, using education level as a control variable.

**Location**	**Age**	
	***R***	***p***
ACR-L	−0.257	**0.027**
ACR-R	−0.240	**0.040**
ALIC-L	**0.001**	0.992
ALIC-R	**0.043**	0.717
BCC	0.091	0.443
CC	**0.010**	0.935
CGC-L	**−0.045**	0.706
CGC-R	0.072	0.539
CGH-L	−0.108	0.360
CGH-R	−0.109	0.357
CR-L	−0.297	**0.010**
CR-R	−0.298	**0.010**
CST-L	**<0.001**	0.999
CST-R	**0.009**	0.942
EC-L	−0.098	0.406
EC-R	−0.092	0.434
FX	−0.215	0.066
FXST-L	**−0.035**	0.766
FXST-R	−0.091	0.440
GCC	−0.179	0.127
IC-L	−0.212	0.070
IC-R	−0.144	0.222
IFO-L	**−0.025**	0.829
IFO-R	**−0.006**	0.957
PCR-L	−0.251	**0.031**
PCR-R	−0.272	**0.019**
PLIC-L	−0.136	0.249
PLIC-R	−0.147	0.211
PTR-L	**−0.470**	**<0.001**
PTR-R	**−0.366**	**0.001**
RLIC-L	−0.344	**0.003**
RLIC-R	−0.236	**0.043**
SCC	0.078	0.509
SCR-L	−0.242	**0.037**
SCR-R	−0.248	**0.033**
SFO-L	–**0.011**	0.926
SFO-R	−0.086	0.467
SLF-L	−0.194	0.098
SLF-R	−0.162	0.168
SS-L	−0.329	**0.004**
SS-R	−0.325	**0.005**
UNC-L	0.271	**0.020**
UNC-R	0.059	0.616

*Significant results are presented in bold case*.

**Figure 2:**
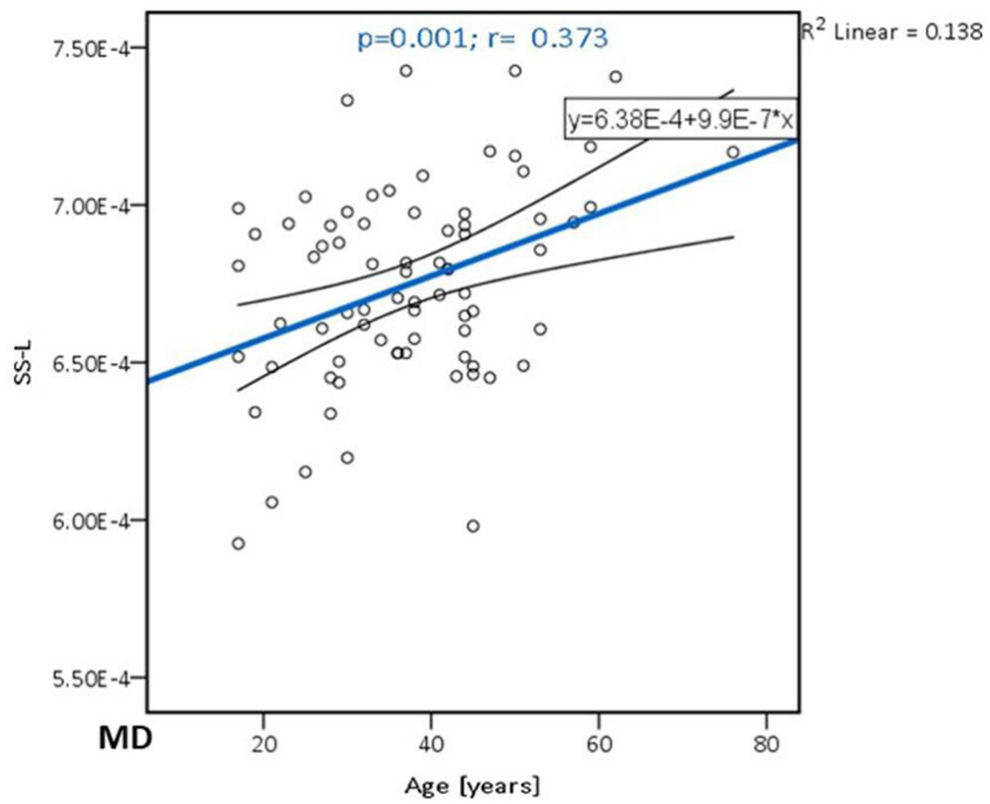
The trend of MD increase with advancing age in the sagittal stratum on the left side.

Table 3 shows the correlations between MD and age and between MD and educational level of the participants are presented for the analyzed locations. A significant positive correlation was confirmed only in SS on the left side (*p* = **0.001**) (Figure 2). There were no significant correlations of this parameter with the level of formal education or the MMSE score. In Table 3A, the results of the partial correlation are summarized.

**Table 3:** Results of Pearson's correlation of MD with age and education level of the study participants in the observed locations (*r*-correlation coefficient).

**Location**	**Age**		**Education level**	
	***r***	***p***	***r***	***p***
ACR-L	0.214	0.066	−0.104	0.375
ACR-R	0.145	0.215	−0.109	0.353
ALIC-L	0.104	0.376	0.090	0.442
ALIC-R	**0.018**	0.881	**0.009**	0.941
BCC	0.232	**0.046**	**0.020**	0.862
CC	0.243	**0.036**	0.092	0.435
CGC-L	0.193	0.098	**0.030**	0.798
CGC-R	0.166	0.154	−0.111	0.343
CGH-L	0.229	**0.048**	−0.053	0.654
CGH-R	0.197	0.091	−0.092	0.430
CR-L	0.278	**0.016**	−0.086	0.462
CR-R	0.228	**0.049**	−0.058	0.619
CST-L	0.207	0.075	−0.067	0.570
CST-R	0.212	0.068	0.058	0.622
EC-L	0.304	**0.008**	**−0.008**	0.946
EC-R	0.304	**0.008**	**0.034**	0.775
FX	0.228	**0.049**	**−0.049**	0.677
FXST-L	**0.014**	0.904	0.119	0.310
FXST-R	0.119	0.308	0.061	0.605
GCC	0.247	**0.032**	−0.061	0.606
IC-L	0.171	0.141	0.184	0.113
IC-R	0.146	0.211	0.122	0.296
IFO-L	0.130	0.267	**0.004**	0.974
IFO-R	0.134	0.252	−0.116	0.321
PCR-L	0.320	**0.005**	0.055	0.640
PCR-R	0.238	**0.040**	0.113	0.335
PLIC-L	0.130	0.265	0.209	0.072
PLIC-R	0.095	0.420	0.215	0.064
PTR-L	0.309	**0.007**	0.152	0.193
PTR-R	0.266	**0.021**	0.176	0.132
RLIC-L	0.234	**0.044**	0.170	0.144
RLIC-R	0.248	**0.032**	0.067	0.567
SCC	0.156	0.180	0.313	**0.006**
SCR-L	0.279	**0.015**	−0.117	0.318
SCR-R	0.277	**0.016**	−0.085	0.468
SFO-L	0.296	**0.010**	−0.090	0.444
SFO-R	0.153	0.189	−0.141	0.229
SLF-L	0.256	**0.027**	**−0.042**	0.719
SLF-R	0.273	**0.018**	**0.010**	0.933
SS-L	**0.372**	**0.001**	**0.016**	0.893
SS-R	0.338	**0.003**	−0.056	0.633
UNC-L	0.068	0.562	0.078	0.507
UNC-R	0.214	0.065	0.239	**0.039**

*Significant results are presented in bold case*.

**Table 3A:** The results of partial correlation of MD with age, using education level as a control variable.

**Location**	**Age**	
	***R***	***p***
ACR-L	0.202	0.084
ACR-R	0.132	0.263
ALIC-L	0.118	0.316
ALIC-R	**0.019**	0.873
BCC	0.237	**0.042**
CC	0.259	**0.026**
CGC-L	0.199	0.089
CGC-R	0.153	0.193
CGH-L	0.224	0.055
CGH-R	0.186	0.112
CR-L	0.270	**0.020**
CR-R	0.223	0.056
CST-L	0.200	0.088
CST-R	0.223	0.057
EC-L	0.306	**0.008**
EC-R	0.312	**0.007**
FX	0.223	0.056
FXST-L	**0.031**	0.791
FXST-R	0.129	0.273
GCC	0.242	**0.038**
IC-L	0.203	0.083
IC-R	0.166	0.157
IFO-L	0.132	0.264
IFO-R	0.120	0.310
PCR-L	0.331	**0.004**
PCR-R	0.258	**0.027**
PLIC-L	0.165	0.160
PLIC-R	0.129	0.274
PTR-L	0.337	**0.003**
PTR-R	0.298	**0.010**
RLIC-L	0.264	**0.023**
RLIC-R	0.261	**0.025**
SCC	0.213	0.069
SCR-L	0.267	**0.021**
SCR-R	0.268	**0.021**
SFO-L	0.288	**0.013**
SFO-R	0.136	0.247
SLF-L	0.253	**0.030**
SLF-R	0.278	**0.017**
SS-L	**0.378**	**0.001**
SS-R	0.334	**0.004**
UNC-L	0.080	0.499
UNC-R	0.258	**0.026**

*Significant results are presented in bold case*.

**Figure 3:**
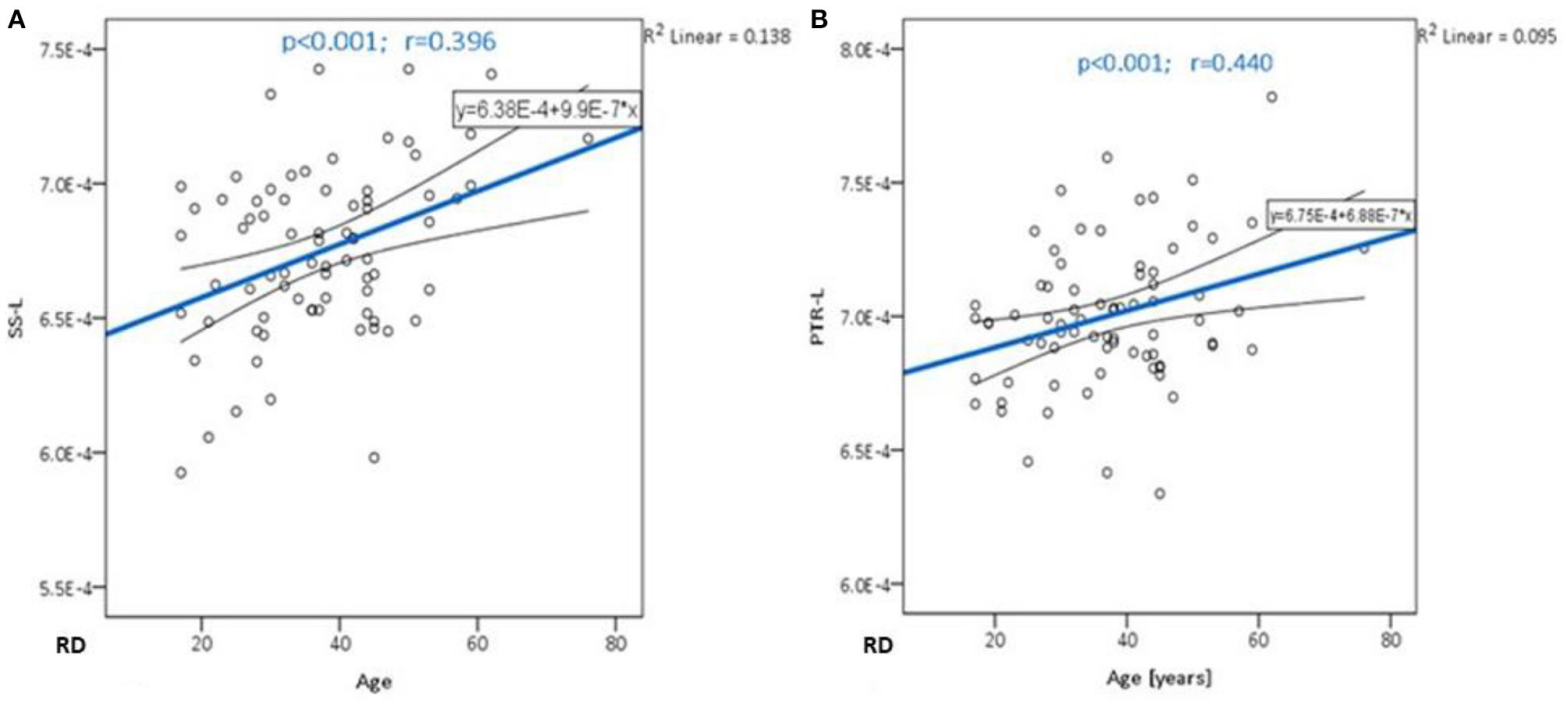
The trend of RD increase with advancing age in the sagittal stratum **(A)** and posterior thalamic radiation **(B)**.

Significant positive correlations were detected between RD and age in PTR (on the left *p*< **0.001** and on the right *p* = **0.001**, Figure 3A), SS (on the left *p*< **0.001**, and on the right *p*< **0.001**, Figure 3B), and in RLIC (on the left *p* = **0.001**). There were no significant correlations of this parameter with the level of formal education or the MMSE score (Table 4). In Table 4A, the results of the partial correlation are summarized.

**Table 4:** Results of Pearson's correlation of RD with age and education level of the study participants in the observed locations (*r*-correlation coefficient).

**Location**	**Age**		**Education level**	
	***r***	***p***	***r***	***p***
ACR-L	0.270	**0.019**	−0.067	0.568
ACR-R	0.242	**0.036**	−0.078	0.507
ALIC-L	0.079	0.498	0.148	0.206
ALIC-R	**0.000**	0.997	0.079	0.503
BCC	**0.037**	0.752	0.143	0.223
CC	0.090	0.443	0.186	0.109
CGC-L	0.116	0.320	**0.034**	0.772
CGC-R	0.116	0.320	−0.070	0.550
CGH-L	0.232	**0.045**	−0.080	0.497
CGH-R	0.228	**0.049**	−0.144	0.218
CR-L	0.347	**0.002**	**−0.044**	0.708
CR-R	0.331	**0.004**	**−0.017**	0.887
CST-L	0.138	0.236	−0.055	0.636
CST-R	0.167	0.153	–**0.038**	0.744
EC-L	0.287	**0.013**	**−0.026**	0.827
EC-R	0.257	**0.026**	**−0.007**	0.952
FX	0.234	**0.044**	−0.072	0.538
FXST-L	0.071	0.545	**0.010**	0.935
FXST-R	0.159	0.172	−0.060	0.612
GCC	0.214	0.065	0.063	0.592
IC-L	0.238	**0.040**	0.163	0.163
IC-R	0.179	0.125	0.115	0.327
IFO-L	**0.038**	0.744	0.070	0.553
IFO-R	0.071	0.546	**−0.031**	0.790
PCR-L	0.357	**0.002**	0.114	0.328
PCR-R	0.309	**0.007**	0.179	0.125
PLIC-L	0.140	0.232	0.206	0.076
PLIC-R	0.126	0.282	0.220	0.058
PTR-L	**0.440**	**<0.001**	0.108	0.357
PTR-R	**0.369**	**0.001**	0.075	0.521
RLIC-L	**0.377**	**0.001**	**0.043**	0.716
RLIC-R	0.303	**0.008**	**−0.019**	0.870
SCC	**0.017**	0.888	0.296	**0.010**
SCR-L	0.334	**0.003**	−0.075	0.521
SCR-R	0.334	**0.003**	**−0.043**	0.711
SFO-L	0.311	**0.007**	−0.102	0.383
SFO-R	0.184	0.114	−0.090	0.440
SLF-L	0.263	**0.023**	**−0.026**	0.825
SLF-R	0.246	**0.033**	**0.028**	0.812
SS-L	**0.396**	**<0.001**	−0.061	0.606
SS-R	**0.392**	**<0.001**	−0.121	0.299
UNC-L	−0.148	0.205	0.225	0.052
UNC-R	**0.043**	0.716	0.339	**0.003**

*Significant results are presented in bold case*.

**Table 4A:** The results of partial correlation of RD with age, using education level as a control variable.

**Location**	**Age**	
	***R***	***p***
ACR-L	0.254	**0.021**
ACR-R	0.241	**0.035**
ALIC-L	0.075	0.480
ALIC-R	**0.001**	0.956
BCC	**0.035**	0.777
CC	0.090	0.445
CGC-L	0.114	0.318
CGC-R	0.112	0.316
CGH-L	0.244	0.051
CGH-R	0.226	**0.048**
CR-L	0.345	0.054
CR-R	0.331	0.053
CST-L	0.141	0.235
CST-R	0.160	0.158
EC-L	0.257	**0.018**
EC-R	0.250	**0.020**
FX	0.234	**0.042**
FXST-L	0.068	0.532
FXST-R	0.159	0.172
GCC	0.202	0.075
IC-L	0.238	0.054
IC-R	0.179	0.125
IFO-L	**0.038**	0.744
IFO-R	0.071	0.546
PCR-L	0.357	0.201
PCR-R	0.309	0.207
PLIC-L	0.287	0.232
PLIC-R	0.185	0.282
PTR-L	0.323	**0.033**
PTR-R	0.356	**0.042**
RLIC-L	0.343	**0.028**
RLIC-R	0.373	0.064
SCC	**0.017**	0.880
SCR-L	0.289	0.052
SCR-R	0.300	0.060
SFO-L	0.222	0.075
SFO-R	0.184	0.114
SLF-L	0.263	**0.023**
SLF-R	0.246	**0.033**
SS-L	0.311	**0.020**
SS-R	0.325	**0.042**
UNC-L	0.140	0.211
UNC-R	**0.043**	0.716

*Significant results are presented in bold case*.

**Figure 4:**
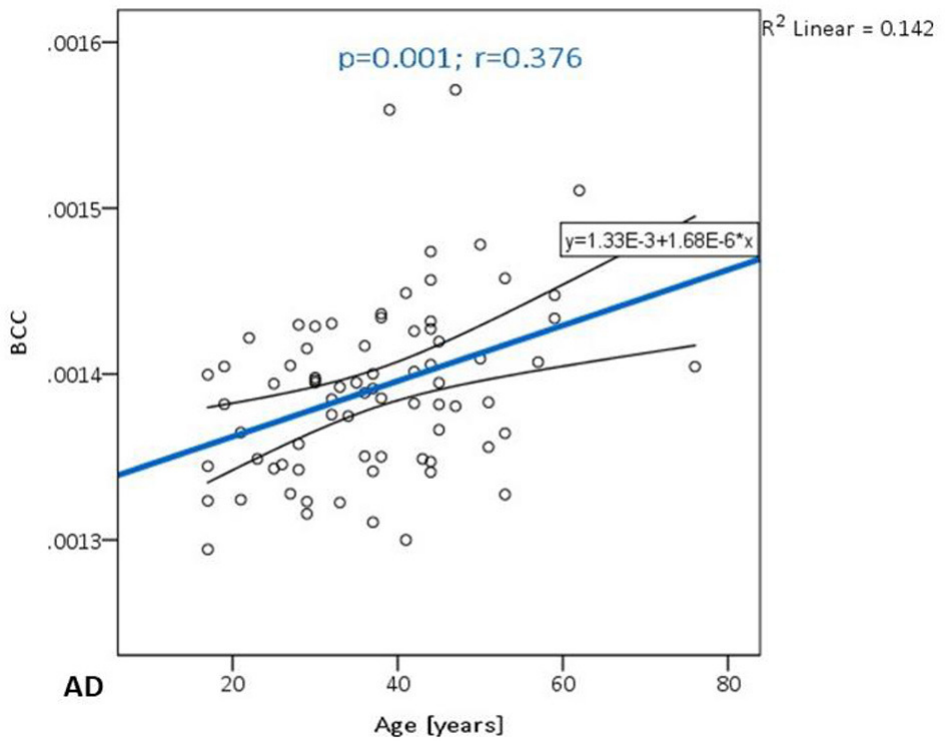
The trend of AD increase in the body of the corpus callosum.

Significant positive correlations between AD and age were detected in BCC (*p* = **0.001**, Figure 4). There were no significant correlations between AD and educational level (Table 5).

**Table 5:** Results of Pearson's correlation of AD with age and education level of the study participants in the observed locations (*r*-correlation coefficient).

**Location**	**Age**		**Education level**	
	***r***	***p***	***r***	***p***
ACR-L	–**0.013**	0.911	−0.102	0.386
ACR-R	−0.097	0.407	−0.089	0.447
ALIC-L	0.084	0.475	**−0.009**	0.936
ALIC-R	**0.028**	0.814	−0.075	0.523
BCC	**0.376**	**0.001**	−0.162	0.166
CC	0.316	**0.006**	−0.088	0.451
CGC-L	0.152	0.192	**0.004**	0.974
CGC-R	0.104	0.374	−0.080	0.497
CGH-L	0.143	0.220	**0.000**	0.997
CGH-R	0.103	0.378	**−0.006**	0.957
CR-L	**0.034**	0.775	−0.108	0.356
CR-R	**−0.038**	0.747	−0.087	0.457
CST-L	0.177	0.130	**−0.046**	0.692
CST-R	0.176	0.132	0.130	0.265
EC-L	0.219	0.059	**0.024**	0.841
EC-R	0.253	**0.029**	0.096	0.410
FX	0.166	0.155	**0.036**	0.757
FXST-L	−0.053	0.652	0.184	0.114
FXST-R	**0.028**	0.813	0.155	0.186
GCC	0.144	0.216	−0.203	0.080
IC-L	**0.039**	0.743	0.144	0.217
IC-R	**0.039**	0.743	0.077	0.514
IFO-L	0.208	0.073	−0.092	0.434
IFO-R	0.142	0.225	−0.166	0.155
PCR-L	0.106	0.367	−0.052	0.657
PCR-R	**0.021**	0.859	**−0.037**	0.751
PLIC-L	0.082	0.484	0.152	0.193
PLIC-R	**0.008**	0.945	0.103	0.381
PTR-L	−0.203	0.081	0.088	0.451
PTR-R	−0.096	0.412	0.171	0.143
RLIC-L	−0.077	0.509	0.226	0.052
RLIC-R	0.061	0.601	0.143	0.222
SCC	0.238	**0.040**	0.155	0.184
SCR-L	**0.038**	0.748	−0.103	0.380
SCR-R	**0.015**	0.898	−0.078	0.507
SFO-L	0.133	0.254	**−0.030**	0.799
SFO-R	**0.020**	0.868	−0.123	0.293
SLF-L	0.075	0.524	**−0.042**	0.721
SLF-R	0.144	0.217	**−0.026**	0.825
SS-L	0.114	0.329	0.139	0.235
SS-R	0.074	0.528	0.071	0.544
UNC-L	0.340	**0.003**	−0.179	0.124
UNC-R	0.287	**0.013**	−0.052	0.659

*Significant results are presented in bold case*.

However, with education level used as a control variable, the significance of the diffusivity decrease with age in the BCC was lost (Table 5A). There were no significant correlations with MMSE scores.

**Table 5A:** The results of partial correlation of AD with age, using education level as a control variable.

**Location**	**Age**	
	***R***	***p***
ACR-L	**−0.028**	0.814
ACR-R	−0.111	0.346
ALIC-L	0.083	0.480
ALIC-R	**0.017**	0.884
BCC	0.362	**0.002**
CC	0.308	**0.008**
CGC-L	0.154	0.189
CGC-R	0.094	0.424
CGH-L	0.145	0.219
CGH-R	0.103	0.381
CR-L	**0.019**	0.875
CR-R	−0.051	0.667
CST-L	0.172	0.143
CST-R	0.198	0.092
EC-L	0.225	0.054
EC-R	0.270	**0.020**
FX	0.173	0.141
FXST-L	**−0.028**	0.814
FXST-R	0.051	0.669
GCC	0.120	0.310
IC-L	0.060	0.610
IC-R	0.050	0.673
IFO-L	0.198	0.091
IFO-R	0.121	0.303
PCR-L	0.100	0.399
PCR-R	**0.016**	0.893
PLIC-L	0.106	0.370
PLIC-R	**0.023**	0.847
PTR-L	−0.193	0.099
PTR-R	−0.074	0.531
RLIC-L	**−0.047**	0.688
RLIC-R	0.083	0.482
SCC	0.265	**0.022**
SCR-L	**0.024**	0.841
SCR-R	**0.004**	0.972
SFO-L	0.131	0.268
SFO-R	**0.002**	0.984
SLF-L	0.070	0.556
SLF-R	0.142	0.227
SS-L	0.136	0.247
SS-R	0.085	0.471
UNC-L	0.323	**0.005**
UNC-R	0.283	**0.015**

*Significant results are presented in bold case*.

The results of permutation tests are provided in Supplementary Material.

## Discussion

The main goal of the study was to correlate white matter DTI anisotropy and diffusivity measures (FA, MD, RD, and AD) in healthy volunteers with chronological age and formal education level. Additionally, we aimed to determine whether the gradient pattern of these associations was consistent with any of the proposed physiological brain aging patterns.

In our study, FA values showed significant reduction with advancing age in the PTR, which is the dorsal part of thalamocortical radiations connecting the thalamus with cortical centers. It consists of fibers that start from the caudal thalamic nuclei (pulvinar and lateral geniculate) *via* the retrolenticular part of the IC toward the parietal and occipital cortices. The function of this tract is primarily somatosensory (comprises parts of visual, gustatory, and auditory tracts) (28, 29). The relationship between the decline in perceptive and cognitive abilities is well-established in the process of aging; however, growing evidence suggests that there is a direct association between sensory deprivation-decreased processing of sensory information- and cognitive decline (30). The decrease in FA was observed in women with anorexia nervosa (31), patients with a high risk of psychosis converting to manifest psychosis (32), methamphetamine addicts (33), and children with cerebral palsy (34). An interesting recent study showed the relationship between cigarette smoking and changes in FA and MD in the PTR (35), supporting the necessity to include the daily habits as a sine-qua-non in future DTI studies (eg., nicotine and alcohol consumption, substance abuse, and pharmaceuticals).

A significant increase in MD with advancing age was observed in the SS on the left side. This tract is positioned in the deep lateral aspect of the cerebral hemisphere and organized into three layers: superficial (consisting of inferior longitudinal fasciculus and inferior part of SLF), middle (fronto- occipital fasciculus), and deep (fibers of optic radiation) (36). It is included in the information transport from parietal, occipital, cingulate, and temporal gyri to the subcortical nuclei (thalamus and pontine nuclei). In the past studies, the increase in MD was detected in patients with essential tremors and has been suggested as one of the differentials from Parkinson's disease in these patients (37). It was also observed in the mild traumatic injury of the brain (38), children with ADHD (associated with aberrant myelination) (39) but was also observed in healthy cigarette smokers (40).

A significant increase in RD with advanced age was detected in the PTR, SS, and RLIC. RLIC is positioned dorsally to the corticospinal and corticopontineus tracts but represents a functionally separate entity because it carries parts of optic radiation, proximal part of PTR, and occipito-tectal and occipito-pontine fibers. Since the increase in this parameter was observed in the PTR and RLIC, it is clear that, along with the close anatomic relationship, there is also a functional connection between these two structures, mainly in the somatosensory information transmission. SS is also functionally associated with the latter two tracts. Disturbances in the diffusivity of RLIC have been priorly observed in depression (41), bipolar disorder (36), methadone addiction (42), and traumatic injury of the brain associated with sports (43).

A significant increase in AD with advancing age was detected in BCC. The corpus callosum is the biggest commissural tract that connects corresponding regions of cerebral hemispheres. Different parts of CC are included in the transmission of various information, with premotor and supplementary motor fibers of the cortex located in the body 409. Madden et al. did not show differences in AD with advanced age (44). However, Fan et al. presented differences in FA between young (20–28 years) and elderly adults (60–75 years) primarily in the anterior portion of CC (within interhemispheric fibers), that were positively correlated with performance on the visuospatial tasks—AD was not explored in that particular study (45). Evidence supporting the “last-in-first-out” theory was also presented in the study of Davis et al. presenting greater age-related differences in RD than in AD in the CC and UNC, thus supporting the theory of myelodegeneration and retrogenesis (46). In our study, however, AD was more sensitive to the changes in these regions, thus indicating that these theoretical interpretations of the white matter diffusivity changes are in fact a simplification of the aging process. That is why we highlight the need for a deeper understanding of the age-related effect on the white matter integrity.

It is interesting to note that FA, MD, and RD were associated with the diffusivity changes in projection tracts (that connect cortical structures with the brainstem, cerebellum, and spinal medulla). On the contrary, the AD was associated with diffusivity changes in commissural tracts, connecting two hemispheres. In a recent longitudinal study, that followed healthy participants aged 50–84 years (at the moment of the first scan, the average age of women was 63.85 years and men was 67.31 years) during the follow-up period of 7 years, showed the most prominent changes in the FA and RD in commissural and projection tracts (47). It is possible that the changes in these two parameters are first observed in projection tracts, as shown in our study, and later during chronological aging, are followed by diffusivity changes in the commissural tracts as well.

Little is known about the oligodendrocyte dynamics and myelin sheath remodeling in the mature brain. However, recent studies confirmed that sensory enrichment could induce experience-dependent myelination and robustly increase the integration of oligodendrocytes (48). Reversely, the results of our study, with age-related changes detected mainly in the somatosensory tracts may imply that somatosensory deprivation during healthy aging negatively affects myelin sheaths (in the sense of myelin degradation and/ or disintegration) and results in DTI metrics changes. This effect of somatosensory deprivation on white matter integrity during the brain aging, should, in our opinion, be included in the current theories of the white matter degradation during aging process, along with the myelodegeneration-retrogenesis and gradients of degenerations (antero- posterior and supero-inferior).

**Figure 5:**
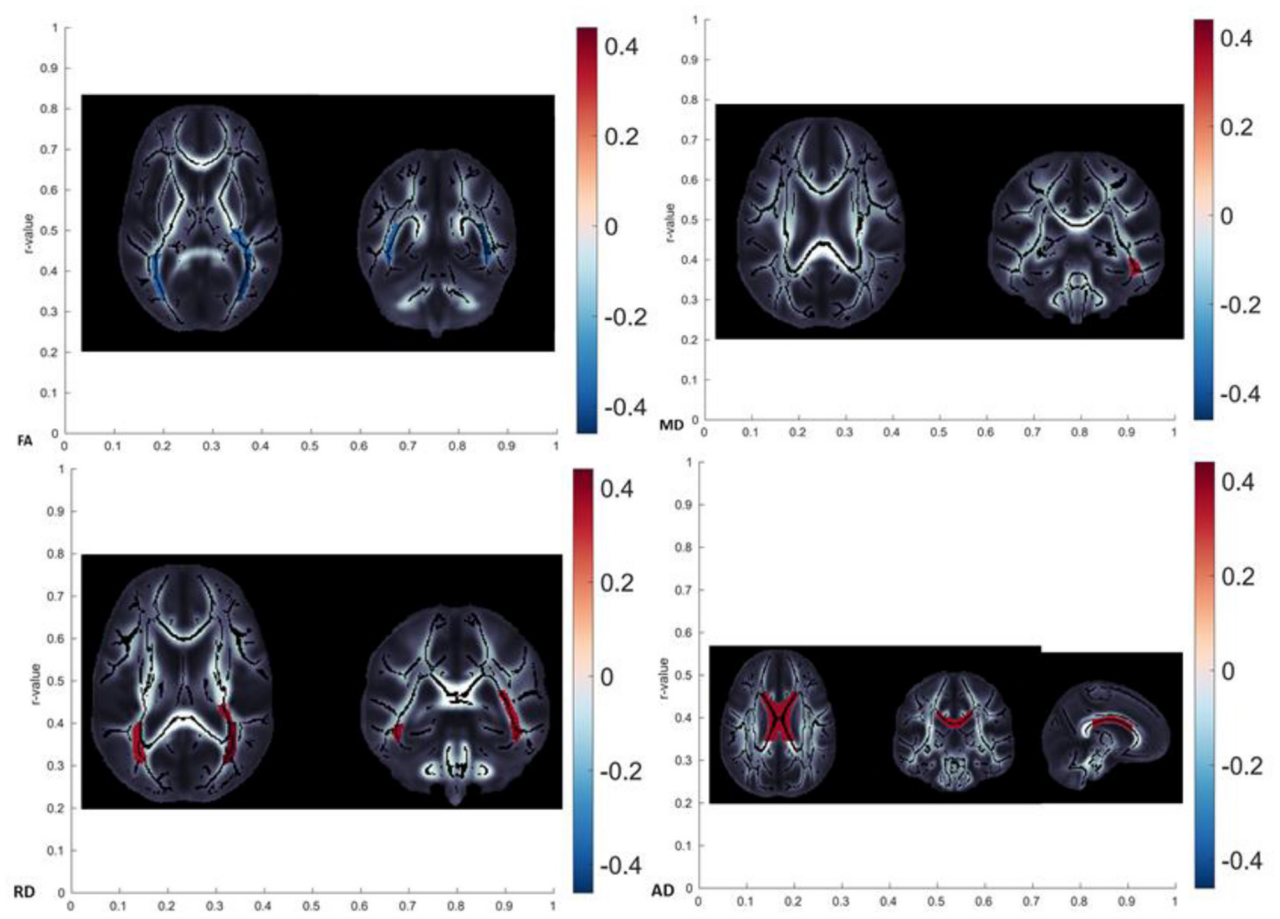
Brain maps showing localization with significant age-related diffusivity changes (FA showed a significant decrease in posterior thalamic radiation, MD showed a significant increase in left sagittal stratum, RD showed a significant increase in posterior thalamic radiation, and AD showed significant changes in the body of corpus callosum).

It is interesting to see the overlap in the DTI metrics changes observed in specific tracts, which could potentially clear the etiology of age-related changes. We tried to group the differences in DTI metrics in the following patterns, as shown in Table 6. In our study, the observed patterns of age-related diffusivity changes were the FA-RD (detected in PTR) and the MD-RD pattern (detected in SS), speaking in favor of myleodegeneration over axonal damage in the named tracts (Figure 5 showing brain maps). In RLIC, we found the diffusivity changes reflected as RD increased, also speaking in favor of myelodegeneration. Given the absence of manifest cognitive impairment and the strict exclusion criteria that we proposed for randomizing the subjects, in our opinion, patterns found in our study sample were expected. In the cohort of healthy, fully functional, and cognitively unchallenged subjects, age-related myelin degeneration and loss seem to be the leading mechanism of white matter diffusivity changes. One recent study also tried to group the findings in DTI metrics across the brain white matter tracts. The FA–RD pattern was observed only in the posterior thalamic radiation in our study sample, while in the study of Molloy et al., it was the most observed pattern, detected in more than 30% of the brain tracts (10). The FA–RD–AD pattern in this study was observed in almost 30% of the brain (10). Other patterns were more uncommon. Burzynska et al. confirmed that FA was significantly correlated with age in more than half of the brain white matter (13). Molloy et al. showed that the highest percentage of voxels significantly correlated with age were found in the forceps minor (part of CC) (10). The FA–MD–RD pattern is related to chronic white matter degeneration, mainly dependent on the myelin loss during the aging process. The changes in AD, however, have been linked to axonal damage (in combination with FA changes representing acute axonal swelling and fragmentation) (13). FA alone changes have been suggested to reflect mild microstructural changes with the loss of fiber structure without great tissue loss. Additional MD changes, following FA changes, reflect the presence of tissue loss and atrophy (46). The changes in RD (combined with FA and, sometimes AD) were related to gliosis and subsequent increase in extracellular tissue (48). Several studies confirmed that greater changes in RD (compared to AD) were found across the brain, similar to our results, suggesting that the leading mechanism of white matter degradation is to be associated with myelin degeneration (10, 13, 17, 46). The findings in AD were inconsistent in our study, similar to other studies from the available literature, showing both positive and negative correlations with age (10, 13, 46). The explanation for this finding in our study might be that our cohort had more prevalent chronic axonal damage (related to the increase in AD) or that these changes can be related to the incoherence in the regions of crossing fibers (which is, in our opinion, a more probable explanation).

**Table 6:** Patterns of significant findings in selected ROIs.

**Location**	**FA**	**MD**	**RD**	**AD**
Posterior thalamic radiation	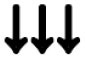		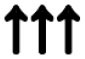	
Sagittal stratum		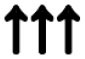	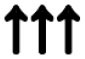	
Retrolenticular internal capsule			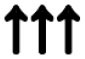	
Body of the corpus callosum				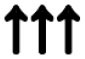

*The arrows represent significant decrease or increase, depending on the direction they are pointing to downward-decrease, upward-increase*.

**Figure 6:**
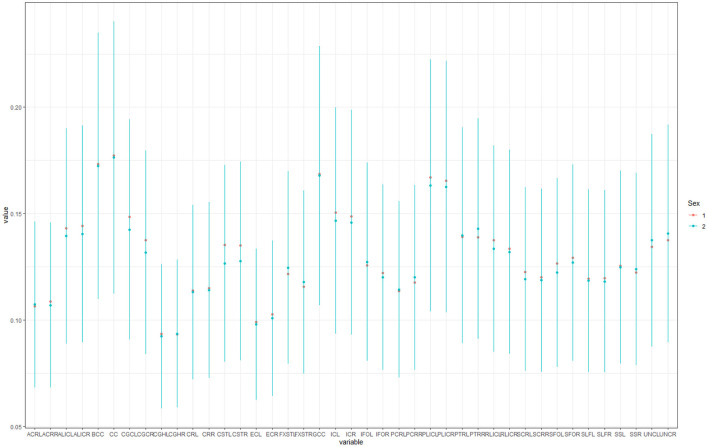
Box plots showing gender differences in observed locations (1-male, 2- female).

Finally, no significant gender differences related to white matter diffusivity metrics were detected (Figure 6). However, this finding might be associated with the small sample with uneven distribution of men and women (males significantly outnumbering females).

Heterogeneity in findings in diffusivity changes across the brain, in our opinion, cannot be explained by one single gradient of physiological brain aging. It seems that different patterns of degradation are true for different fiber tracts in the brain and that no currently available theory can globally explain age-related changes in the brain. Furthermore, the effect of somatosensory decline should be included as one of the important covariables in the existing patterns.

In addition, a recent study by Behler et al. showed that diffusion properties of most white matter tracts in a healthy aging brain followed a triphasic pattern (in a simplified manner, FA showed a gradual increase during early adulthood followed by a stable state during middle age, and gradual decline in senium; RD, MD, and AD showed a decline during the early adulthood, followed by the stable state in middle age and increase in senium), so that non-linear age correction should be applied to most of the tracts. Cerebellar tracts (superior and middle cerebellar pedunculi) showed basically no change over time (i.e., age correction is not applicable); optic radiation and anterior limb of the internal capsule showed linear regression with age, meaning that non-linear age correction is not necessary (49).

### Study Limitations

There are some limitations to the study performed. Even though the number of participants was justified by the power analysis, it is possible that some correlations would have shown to be significant if the study sample were larger, and this might be resolved in a future study. However, it is important to highlight that the exclusion criterion for our study sample was the presence of white matter lesions including white matter hyperintensities (WMH) which represents a pertinent factor for the assessment of the changes in the white matter related to aging.

The second limitation of the study was the lack of a detailed neurocognitive assessment of our participants (only a screening cognitive test was performed as a part of the exclusion criteria). MMSE scoring test represents a screening test, of high clinical importance, but is not sensitive enough for the cases of early cognitive impairment, thus, in our opinion, suitable for the exclusion of cognitively challenged persons, but not suitable for detailed correlations. Some of the tracts we analyzed are known to be associated with affect and personality features, which means that additional assessment of depression and anxiety would be recommendable.

An additional limitation is a cross-sectional evaluation and the lack of longitudinal design, since the changes in diffusivity measurements may follow certain trajectories during chronological aging in an individual.

It is necessary to point out that the maturation of the fibers does not follow the identical dynamics in all observed tracts. Some of the tracts (IFO, AIC, PLIC, SCC, BCC, GCC, UNC, SFO, IFO, SLF) reach the full maturity (maximal FA and minimal MD values) between 20 and 40 years of age (50). In one part of our study sample, these tracts potentially did not develop their full maturity, given that the average age of the sample was around 37 years.

The final (small) limitation of the study is the lack of information on prematurity, which could also influence the changes in tract diffusivity (CC in the first place) (51).

Even though there were no significant comorbidities noted in the study sample, it will be reasonable to include additional information on metabolic function (blood glucose level, lipidemia, parameters of liver function, arterial tension, etc.) and lifestyle in the future studies (52, 53).

It is also worth mentioning that advanced techniques for evaluation of DWI data could provide more detailed information than the DTI option (54) and added to the potential of longitudinal studies in revealing the gradients of changes in the DTI metrics are two main recommendations for the future studies.

## Conclusion

All four DTI metrics showed significant correlations with the advancing age of healthy participants (FA in posterior thalamic radiation, MD in left sagittal stratum, RD in the posterior thalamic radiation, sagittal stratum, and retrolenticular limb of internal capsule, and AD in body of the corpus callosum). According to our study, RD showed the richest correlations with age, out of all DTI metrics. FA, MD, and RD showed significant changes in the diffusivity of projection fibers, while AD presented diffusivity changes in the commissural fibers.

It is important to realize that all four DTI metrics are necessary for the assessment of healthy brain aging effect on white matter integrity, given that routinely analyzed parameters (FA and MD) do not allow complete insight into diffusivity changes. Since the changes in the white matter integrity that we observed during healthy aging also overlap with changes observed in several pathological conditions—psychiatric disorders, traumatic lesions, and dementia/MCI—caution is necessary when interpreting these differences in a healthy individual. In such cases, a longitudinal follow-up is necessary to track the trajectories of degenerative changes associated with the aging brain.

Finally, the observed heterogeneity in diffusivity changes across the brain, in our opinion, cannot be explained by a single aging pattern (antero-posterior, supero-inferior, or myelodegeneration- retrogenesis). It seems that different patterns of degradation are true for different fiber tracts in the brain and that no currently available theory can globally explain age-related changes in the brain. Some additional factors, such as the effect of somatosensory decline, should be included as one of the important covariables in the existing patterns.

## Data Availability Statement

The raw data supporting the conclusions of this article will be made available by the authors, withoutundue reservation.

## Ethics Statement

The studies involving human participants were reviewed and approved by Ethical Committee of Faculty of Medicine University of Novi Sad. The patients/participants provided their written informed consent to participate in this study.

## Author Contributions

JB drafted the article. JB, MT, NB, DL, NJ, and TN were involved in acquisition, collection, and analysis of the data. DK, MT, and ML performed critical revision of intellectual content. All authors saw and approved the final version of drafted manuscript.

## Funding

TN and NJ were funded, in part, by NIH grants: R01 AG059874, U54 EB020403, T32 AG058507, and U01 AG068057. The authors also received partial grant support from Biogen, Inc., for research unrelated to this work. JB, DK, and DL were funded by Provincial Secretariat for Higher Education and scientific Research, grant No. 142-451-2600/2021-01.

## Conflict of Interest

The authors declare that the research was conducted in the absence of any commercial or financial relationships that could be construed as a potential conflict of interest.

## Publisher's Note

All claims expressed in this article are solely those of the authors and do not necessarily represent those of their affiliated organizations, or those of the publisher, the editors and the reviewers. Any product that may be evaluated in this article, or claim that may be made by its manufacturer, is not guaranteed or endorsed by the publisher.

## References

[1] Ishii R Canuet L Aoki Y Hata M Iwase M Ikeda S. Healthy and pathological brain aging: from the perspective of oscillations, functional connectivity and signal complexity. Neuropsychology. (2017) 75:151–61. 10.1159/000486870 29466802

[2] Desai AK Grossberg GT Chibnall JT. Healthy brain aging: a road map. Clin Geriatr Med. (2010) 26:1–16. 10.1016/j.cger.2009.12.002 20176289

[3] Huisman TAGM. Diffusion-weighted and diffusion tensor imaging of the brain made easy. Cancerimaging. (2010) 10: S163–71. 10.1102/1470-7330.2010.9023 20880787 PMC2967146

[4] Drake-Perez M Boto J Fitsiori A Lovblad K Vargas MI. Clinial applications of diffusion weighted imaging in neuroradiology. Insights Imaging. (2018) 9:535–47. 10.1007/s13244-018-0624-3 29846907 PMC6108979

[5] O'Donnell LJ Westin CF. An introduction to diffusion tensor image analysis. Neurosurg Clin N Am. (2011) 22:185–VII. 10.1016/j.nec.2010.12.004 21435570 PMC3163395

[6] O'Sullivan M Jones DK Summers PE Morris RG Williams SC Markus HS. Evidence for cortical “disconnection” as a mechanism of age-related cognitive decline. Neurology. (2001) 57:632–8. 10.1212/WNL.57.4.632 11524471

[7] Andrews-Hanna JR Snyder AZ Vincent JL Lustig C Head D Raichle ME Buckner RL. Disruption of large-scale brain systems in advanced aging. Neuron. (2007) 56:924–35. 10.1016/j.neuron.2007.10.038 18054866 PMC2709284

[8] Salat DH. The declining infrastructure of the aging brain. Brain Connect. (2011) 1:279–93. 10.1089/brain.2011.0056 22432418 PMC3621330

[9] Vinke EJ de Groot M Venkatraghavan V Klien S Niessen WJ Ikram MA. Trajectories of imaging markers in brain aging: the Rotterdam study. Neurobiol Aging. (2018) 71:32–40. 10.1016/j.neurobiolaging.2018.07.001 30077040

[10] Molloy CJ Nugent S Bokde ALW. Alterations in diffusion measures of white matter integrity associated with healthy aging. J Gerontol A Bio Sci Med Sci. (2019) 22:945–54. 10.1101/540443 31830253

[11] Wasenaar TM Yaffe C van der Werf YD Sexton C. Associations between modifiable risk factors and white matter of the aging brain: insights from diffusion tensor imaging studies. Neurobiol Aging. (2019) 80:56–70. 10.1016/j.neurobiolaging.2019.04.006 31103633 PMC6683729

[12] Brickman AM Meier IB Korgaonkar MS Provenzano FA Grieve SM Siedlecki KL. Testing the white matter retrogenesis hypothesis of cognitive aging. Neurobiol Aging. (2012) 33:1699–715. 10.1016/j.neurobiolaging.2011.06.001 21783280 PMC3222729

[13] Burzynska AZ Preuschhof C Backman L Nyberg L Li SC Lindenberger U. Age-related differences in white matter microstructure: region-sepcific patterns of diffusivity. Neuroimage. (2010) 49:2104–12. 10.1016/j.neuroimage.2009.09.041 19782758

[14] Mayo CD Garcia-Barrera MA Mazerolle EL Ritchie LJ Fisk JD Gawryluk JR. Relationship between DTI metrics and cognitive function in Alzheimer's disease. Front Aging Neurosci. (2019) 10:436. 10.3389/fnagi.2018.00436 30687081 PMC6333848

[15] Ashrafi MR Amanat M Garshasbi M Kameli R Nilipour Y Heidari M. An update on clinical, pathological, diagnostic and therapeutic perspectives of childhood leukodystrophies. Expert Rev Neurother. (2020) 20:65–84. 10.1080/14737175.2020.1699060 31829048

[16] Bach M Laun FB Leemans A Tax CMW Biessels GJ Stieltjes B. Methodological considerations on tract-based spatial statistics (TBSS). Neuroimage. (2014) 100:358–69. 10.1016/j.neuroimage.2014.06.021 24945661

[17] Bennett IJ Madden DJ Vaidya CJ Howard DV Howard JH Jr. Age-related differencesin multiple measures of white matter integrity: a diffusion tensor imaging study of healthy aging. Hum Brain Map. (2010) 31:378–90. 10.1002/hbm.20872 19662658 PMC2826569

[18] Zahr NM Rohlfing T Pfefferbaum A Sullivan EV. Problem solving, working memory, and motor correltaes of association and commissural fiber bundles in normal aging: a quantitative fiber tracking study. Neuroimage. (2009) 44:1050–62. 10.1016/j.neuroimage.2008.09.046 18977450 PMC2632960

[19] Tian L Ma L. Microstructural changes of the human brain from early to mid-adulthood. Front Hum Neurosci. (2017) 11:393. 10.3389/fnhum.2017.00393 28824398 PMC5545923

[20] Schmierer K Scaravilli F Altmann DR Barker GJ Miller DH. Magnetization transfer ratio and myelin in postmortem multiple sclerosis brain. Ann Neurol. (2004) 56:407–15. 10.1002/ana.20202 15349868

[21] Folstein MF Folstein SE McHugh PR. “Mini-mental state”. A practical method for grading the cognitive state of patients for clinician. J Psychiatr Res. (1975) 12:189–98. 10.1016/0022-3956(75)90026-6 1202204

[22] Cavill S Bryden P. Development of handedness: comparison of questionnaire and performance-based measures of preference. Brain Cogn. (2003) 53:149–51. 10.1016/S0278-2626(03)00098-8 14607136

[23] Lapham SC Skipper BJ Owen JP Kleyboecker K Teaf D Thompson B. Alcohol abuse screening instruments: normative test data collected from a first DWI offender screening programe. J Stud Alcohol. (1995) 56:51–9. 10.15288/jsa.1995.56.51 7752633

[24] Basser PJ Mattiello J LeBihan D. MR diffusion tensor spectroscopy and imaging. Biophys J. (1994) 66:259–67. 10.1016/S0006-3495(94)80775-1 8130344 PMC1275686

[25] Jahanshad N Kochunov PV Sprooten E Mandl RC Nichols TE Almasy L. Multi-site genetic analysis of diffusion images and voxelwise heritability analysis: a pilot project of the ENIGMA-DTI working group. Neuroimage. (2013) 81:455–69. 10.1016/j.neuroimage.2013.04.061 23629049 PMC3729717

[26] Avants BB Tustison NJ Song G Cook PA Klein A Gee JC. A reproducible evaluation of ANTs similarity metric performance in brain image registration. Neuroimage. (2011) 54:2033–44. 10.1016/j.neuroimage.2010.09.025 20851191 PMC3065962

[27] Wakana S Jiang H Nagae-Poetscher LM Van Zijl PCM Mori S. Fiber tract-based atlas of human white matter anatomy. Radiology. (2004) 230:77–87. 10.1148/radiol.2301021640 14645885

[28] Hua K Zhang J Wakana S Jiang H Li X Reich DS. Tract probability maps in stereotaxic spaces: analyses of white matter anatomy and tract-specific quantification. Neuroimage. (2008) 39:336–47. 10.1016/j.neuroimage.2007.07.053 17931890 PMC2724595

[29] Aralasmak A Ulmer JL Kocak M Salvan CV Hillis AE Yousem DM. Association, commissural, and projection pathways and their functional deficit reported in literature. J Comput Assist Tomogr. (2006) 30:695–715. 10.1097/01.rct.0000226397.43235.8b 16954916

[30] Roberts KL Allen HA. Perception and cognition in the ageing brain: a brief review of the short- and long-term links between perceptual and cognitive decline. Front Aging Neurosci. (2016) 8:39. 10.3389/fnagi.2016.00039 26973514 PMC4772631

[31] Freiling H Fischer J Wilhelm J Jiang H Li X Reich DS. Microstructural abnormalities of the posterior thalamic radiation and the mediodorsal thalamic nuclei in females with anorexia nervosa- a voxel based diffusion tensor imaging (DTI) study. J Psychiatr Res. (2012) 46:1237–42. 10.1016/j.jpsychires.2012.06.005 22770509

[32] Leon-Ortiz P Reyes-Madrigal F Kochunov P Gomez-Cruz G Moncada-Habib T Malacara M. White matter alterations and the conversion to psychosis: a combined diffusion tensor imaging and glutamate 1H-MRS study. Schizofrenia Res. (2020) S0920–9964(20)30358-3. 10.1016/j.schres.2020.06.006 32595100 PMC10025976

[33] Huang S Yang W Luo J Yan C Liu J. White matter abnormalities based on TBSS and its correlation with impulsivitiy behavior of methamphetamine addicts. Front Psychiatry. (2020) 11:452. 10.3389/fpsyt.2020.00452 32528325 PMC7253705

[34] Hoon AH Jr Stashinko EE Nagae LM Nagae LM Lin DMD Keller J. Sensory and motor deficits in children with cerebral palsy born preterm correlate with diffusion tensor imaging abnormalities in thalamocortical pathways. Dev Med Child Neurol. (2009) 51:697–704. 10.1111/j.1469-8749.2009.03306.x 19416315 PMC2908264

[35] Gray JC Thompson M Bachman C Owens MM Murphy M Palmer R. Associations of cigarette smoking with gray and white matter in the UK Biobank. Neuropsychopharmacology. (2020) 45:1215–22. 10.1038/s41386-020-0630-2 32032968 PMC7235023

[36] Di Carlo DT Benedetto N Duffau H Cagnazzo F Weiss A Castagna M. Microsurgical anatomy of the sagittal stratum. Acta Neurochir. (2019) 161:2319–27. 10.1007/s00701-019-04019-8 31363919

[37] Juttukonda MR Franco G Englot DJ Lin YC Petersen KJ Trujillo P. White matter differences between essential tremor and Parkinson disease. Neurology. (2019) 92:e30–9. 10.1212/WNL.0000000000006694 30504432 PMC6336163

[38] Zhu J Ling J Ding N. Association between diffusion tensor imaging findings and cognitive outcomes following mild traumatic brain injury: a PRISMA-compliant meta-analysis. ACS Chem Neurosci. (2019) 10:4864–9. 10.1021/acschemneuro.9b00584 31746583

[39] Onnink AMH Zwiers MP Hoogman M Mostert JC Dammers J Kan CC. Deviant white matter structure in adults with attentiondeficit/hyperactivity disorder points to aberrant myelination and affects neuropsychological performance. Prog Neuropsychopharmacol Biol Psychiatry. (2015) 63:14–22. 10.1016/j.pnpbp.2015.04.008 25956761 PMC4515357

[40] Liang H Chang L Chen R Oishi K Ernst T Independent and cobined effects of chronic HIV-infection and tobacco smoking on brain microstructure. J Neuroimmune Pharmacol. (2018) 13:509–22. 10.1007/s11481-018-9810-9 30225549 PMC6247419

[41] Hyett MP Perry A Breakspear M Wen W Parker GB. White matter alterations in the internal capsule and psychomotor impairment in melancholic depression. PLoS ONE. (2018) 13:e0195672. 10.1371/journal.pone.0195672 29672517 PMC5908181

[42] Bauer IE Ouyang A Mwangi B Sanches M Zunta-Soares GB Keefe RS. Reduced white matter integrity and verbal fluency impairment in young adults with bipolar disorder: a diffusion tensor imaging study. J Psychiatr Res. (2015) 62:115–22. 10.1016/j.jpsychires.2015.01.008 25684152 PMC4355300

[43] Li W Zhu J Li Q Ye J Chen J Liu J. Brain white matter integrity in heroin addicts during methadonema intenance treatment is related to relapse propensity. Brain Behav. (2016) 6:e00436. 10.1002/brb3.436 27110449 PMC4834937

[44] Madden DJ Spaniol J Costello MC Bucur B White LE Cabeza R. Cerebral white matter integrity mediates adult age differencesin cognitive performance. J Cogn Neurosci. (2009) 21:289–302. 10.1162/jocn.2009.21047 18564054 PMC2676336

[45] Fan YT Fang YW Chen YP Leshikar ED Lin CP Tzeng OJL. Aging, cognition, and the brain: effects of age-related variation in white matter integrity on neuropsychological function. Aging Mental Health. (2019) 23:831–9. 10.1080/13607863.2018.1455804 29634290

[46] Zhang Y Du AT Hayasaka S Jahng GH Hlavin J Zhan W. Patterns of age-related water diffusion changes in human brain by concordance and disconcordance analysis. Neurobiol Aging. (2010) 31:1991–2001. 10.1016/j.neurobiolaging.2008.10.009 19036473 PMC2888604

[47] Bender AR Völkle MC Raz N. Differential aging of cerebral white matter in middle-aged and older adults: a seven-year follow-up. Neuroimage. (2016) 125:74–83. 10.1016/j.neuroimage.2015.10.030 26481675 PMC4691398

[48] Hughes EG Orthmann-Murohy JL Langseth AJ Bergles DE. Myelin remodeling through experiencedependent oligodendrogenesis in the adult somatosensory cortex. Nat Neurosci. (2018) 21:696–706. 10.1038/s41593-018-0121-5 29556025 PMC5920726

[49] Behler A Kassubek J Muller HP. Age-related alterations in DTI metrics in the human brainconsequences for age correction. Front Aging Neurosci. (2021) 6:94. 10.3389/fnagi.2021.682109 34211389 PMC8239142

[50] Olson IR Von Der Heide RJ Alm KH Vyas G. Development of the uncinate fasciculus: implicationsfor theory and developmental disorders. Dev Cogn Neurosci. (2015) 14:50–61. 10.1016/j.dcn.2015.06.003 26143154 PMC4795006

[51] Shim SY Jeong HJ Son DW Jeong JS Oh SH Park SY. Altered microstructure of white matter except the corpus callosum is independent of prematurity. Neonatology. (2012) 102:309–15. 10.1159/000341867 22986463

[52] Hofer S Frahm J. Topography of the human corpus callosum revisited: comprehensive fiber tractography using diffusion tensor magnetic resonance imaging. Neuroimage. (2006) 32:989–94. 10.1016/j.neuroimage.2006.05.044 16854598

[53] Smith JC Lancaster MA Nielson KA Woodard JL Seidenberg M Durgerian S. Interactive effects of physical activity and APO-ξ4 on white matter tract diffusivity in healthy elders. Neuroimage. (2016) 131:102–12. 10.1016/j.neuroimage.2015.08.007 26265157 PMC4746115

[54] Murugavel M Cubon V Putukian M Echemendia R Cabrera J Osherson D. A longitudinal diffusion tensor imaging study assessingwhite matter fiber tracts after sports-related concussion. J Neurotrauma. (2014) 31:1860–71. 10.1089/neu.2014.3368 24786666 PMC4224056

