# Supplementary material for: Gradient Patterns of Age-Related Diffusivity Changes in Cerebral White Matter
Source: Front Neurol. 2022 Jun 2;13:870909. doi: 10.3389/fneur.2022.870909 (PMC9201287; doi:10.3389/fneur.2022.870909)
Supplement: Supplementary file 1 [file Table_1.DOCX]

Permutation test

Supplementary Table 1.Results of permutation test for Pearson’s correlation (age and localization); p-value estimated through 20000 permutations.

|  | MD | FA | L1 | RA |
| --- | --- | --- | --- | --- |
| ACRL | 0.06505 | **0.02**415 | 0.9092 | 0.0659 |
| ACRR | 0.2144 | **0.03**175 | 0.40865 | 0.2163 |
| ALICL | 0.37665 | 0.8415 | 0.4741 | 0.3778 |
| ALICR | 0.87885 | 0.61085 | 0.81415 | 0.881 |
| BCC | **0.04**825 | 0.3215 | **0.00**095 | **0.04**72 |
| CC | **0.03**725 | 0.7388 | **0.00**585 | **0.03**42 |
| CGCL | 0.09795 | 0.7456 | 0.19265 | 0.09835 |
| CGCR | 0.15255 | 0.5005 | 0.36875 | 0.1534 |
| CGHL | **0.04**905 | 0.3289 | 0.221 | **0.04**695 |
| CGHR | 0.08845 | 0.26935 | 0.3741 | 0.0897 |
| CRL | **0.01**71 | **0.01**085 | 0.77055 | **0.01**59 |
| CRR | **0.04**865 | **0.01**09 | 0.7442 | **0.04**69 |
| CSTL | 0.0759 | 0.9863 | 0.12925 | 0.07385 |
| CSTR | 0.0681 | 0.96895 | 0.1296 | 0.06665 |
| ECL | **0.00**835 | 0.38035 | 0.0584 | **0.00**92 |
| ECR | **0.00**94 | 0.39075 | **0.02**97 | **0.00**845 |
| FX | **0.04**925 | **0.04**29 | 0.1522 | **0.04**94 |
| FXSTL | 0.90605 | 0.67995 | 0.651 | 0.9034 |
| FXSTR | 0.30665 | 0.35195 | 0.8118 | 0.31315 |
| GCC | **0.03**535 | 0.17275 | 0.21945 | **0.03**235 |
| ICL | 0.142 | 0.09195 | 0.73515 | 0.14025 |
| ICR | 0.2146 | 0.2606 | 0.7391 | 0.20765 |
| IFOL | 0.27 | 0.99015 | 0.0745 | 0.265 |
| IFOR | 0.2466 | 0.9887 | 0.22375 | 0.25315 |
| PCRL | **0.00**485 | **0.04**875 | 0.37 | **0.00**555 |
| PCRR | **0.04**135 | **0.04**845 | 0.85935 | **0.04**025 |
| PLICL | 0.26285 | 0.3376 | 0.4842 | 0.2663 |
| PLICR | 0.42045 | 0.29885 | 0.94535 | 0.41945 |
| PTRL | **0.00**655 | 0 | 0.0807 | **0.00**66 |
| PTRR | **0.02**055 | **0.00**125 | 0.411 | **0.02**025 |
| RLICL | **0.04**43 | **0.00**27 | 0.51115 | **0.04**485 |
| RLICR | **0.03**275 | **0.03**285 | 0.59635 | **0.03**285 |
| SCC | 0.18135 | 0.353 | **0.04**035 | 0.18265 |
| SCRL | **0.01**475 | **0.03**535 | 0.7444 | **0.01**445 |
| SCRR | **0.01**645 | **0.03**17 | 0.89835 | **0.01**51 |
| SFOL | **0.00**935 | 0.9921 | 0.2507 | **0.00**925 |
| SFOR | 0.18505 | 0.4002 | 0.86155 | 0.18995 |
| SLFL | **0.02**67 | 0.10155 | 0.5283 | **0.02**585 |
| SLFR | **0.01**93 | 0.1786 | 0.2219 | **0.01**675 |
| SSL | **0.00**125 | **0.00**305 | 0.3246 | **0.00**095 |
| SSR | **0.00**32 | **0.00**3 | 0.5291 | **0.00**325 |
| UNCL | 0.56255 | **0.00**995 | **0.00**245 | 0.5625 |
| UNCR | 0.06425 | 0.39055 | **0.01**32 | 0.06685 |

Supplementary Table 2. Results of permutation test for Pearson’s correlation (education and localization); p-value estimated through 20000 permutations.

|  | MD | FA | L1 | RA |
| --- | --- | --- | --- | --- |
| ACRL | 0.37185 | 0.8401 | 0.3882 | 0.37555 |
| ACRR | 0.35635 | 0.6763 | 0.4517 | 0.3601 |
| ALICL | 0.44265 | 0.17705 | 0.9361 | 0.4466 |
| ALICR | 0.9381 | 0.2679 | 0.5275 | 0.94085 |
| BCC | 0.8618 | 0.0914 | 0.1633 | 0.8594 |
| CC | 0.43845 | 0.0637 | 0.4568 | 0.43075 |
| CGCL | 0.7996 | 0.69385 | 0.9736 | 0.7954 |
| CGCR | 0.34505 | 0.6461 | 0.48845 | 0.34455 |
| CGHL | 0.64725 | 0.63425 | 0.99595 | 0.6517 |
| CGHR | 0.4284 | 0.1712 | 0.95725 | 0.4284 |
| CRL | 0.46355 | 0.99155 | 0.3652 | 0.4629 |
| CRR | 0.62425 | 0.9211 | 0.4534 | 0.62015 |
| CSTL | 0.57745 | 0.90655 | 0.69275 | 0.5763 |
| CSTR | 0.62625 | 0.43325 | 0.26665 | 0.6268 |
| ECL | 0.94405 | 0.7468 | 0.83785 | 0.94525 |
| ECR | 0.7765 | 0.53225 | 0.40815 | 0.77505 |
| FX | 0.6786 | 0.1104 | 0.7605 | 0.67875 |
| FXSTL | 0.3134 | 0.40165 | 0.1212 | 0.31125 |
| FXSTR | 0.6074 | 0.1908 | 0.18915 | 0.6019 |
| GCC | 0.608 | 0.3131 | 0.07995 | 0.60795 |
| ICL | 0.11185 | 0.45735 | 0.21885 | 0.11435 |
| ICR | 0.2974 | 0.50125 | 0.52295 | 0.2932 |
| IFOL | 0.97625 | 0.10745 | 0.43475 | 0.97625 |
| IFOR | 0.3224 | 0.6222 | 0.15485 | 0.32475 |
| PCRL | 0.6418 | 0.21695 | 0.6585 | 0.637 |
| PCRR | 0.33915 | **0.04**3 | 0.7522 | 0.33275 |
| PLICL | 0.0717 | 0.2096 | 0.19455 | 0.0735 |
| PLICR | 0.062 | 0.16495 | 0.38015 | 0.06415 |
| PTRL | 0.19265 | 0.6288 | 0.4537 | 0.19205 |
| PTRR | 0.1324 | 0.8619 | 0.1449 | 0.13295 |
| RLICL | 0.14595 | 0.4752 | 0.0502 | 0.1422 |
| RLICR | 0.56765 | 0.39755 | 0.22185 | 0.5705 |
| SCC | **0.00**58 | **0.04**735 | 0.187 | **0.00**49 |
| SCRL | 0.32 | 0.7935 | 0.3758 | 0.32065 |
| SCRR | 0.4696 | 0.87495 | 0.51155 | 0.4651 |
| SFOL | 0.4453 | 0.47325 | 0.7981 | 0.44425 |
| SFOR | 0.22815 | 0.4018 | 0.30095 | 0.2319 |
| SLFL | 0.71715 | 0.85475 | 0.7207 | 0.7228 |
| SLFR | 0.93345 | 0.768 | 0.82525 | 0.9368 |
| SSL | 0.894 | 0.29945 | 0.2373 | 0.88995 |
| SSR | 0.6309 | 0.17235 | 0.54645 | 0.63035 |
| UNCL | 0.50845 | **0.01**505 | 0.1224 | 0.5049 |
| UNCR | **0.03**745 | **0.00**475 | 0.65765 | **0.03**93 |

Supplementary Table 3. Results of permutation test for partial correlations (age and localization, with education as control variable).

|  | MD | FA | L1 | RA |
| --- | --- | --- | --- | --- |
| ACRL | 0.0886 | **0.03**55 | 0.7852 | 0.0943 |
| ACRR | 0.2162 | **0.03**87 | 0.2747 | 0.2202 |
| ALICL | 0.3579 | 0.9914 | 0.432 | 0.3534 |
| ALICR | 0.8901 | 0.7349 | 0.8691 | 0.8932 |
| BCC | 0.0671 | 0.4669 | **0.00**12 | 0.0596 |
| CC | **0.04**14 | 0.9394 | **0.00**55 | **0.04**01 |
| CGCL | 0.1177 | 0.7634 | 0.2024 | 0.1247 |
| CGCR | 0.2536 | 0.5743 | 0.3938 | 0.2617 |
| CGHL | 0.104 | 0.4091 | 0.1991 | 0.1049 |
| CGHR | 0.1537 | 0.2824 | 0.3508 | 0.1489 |
| CRL | **0.03**66 | **0.01**67 | 0.8608 | **0.03**98 |
| CRR | 0.0697 | **0.01**54 | 0.6539 | 0.0739 |
| CSTL | 0.1011 | 0.9986 | 0.1684 | 0.1024 |
| CSTR | 0.0952 | 0.9415 | 0.1328 | 0.0973 |
| ECL | **0.01**81 | 0.3977 | **0.03**46 | **0.01**58 |
| ECR | **0.02**21 | 0.4243 | **0.02**25 | **0.02**3 |
| FX | **0.04**69 | 0.0691 | 0.1671 | 0.0502 |
| FXSTL | 0.805 | 0.7261 | 0.8053 | 0.8107 |
| FXSTR | 0.2909 | 0.3667 | 0.6174 | 0.2927 |
| GCC | 0.0541 | 0.1758 | 0.2757 | 0.0519 |
| ICL | 0.122 | 0.0612 | 0.6127 | 0.1253 |
| ICR | 0.2259 | 0.207 | 0.6468 | 0.231 |
| IFOL | 0.3411 | 0.8504 | 0.1129 | 0.3407 |
| IFOR | 0.3418 | 0.9532 | 0.2978 | 0.3464 |
| PCRL | **0.01**66 | **0.04**01 | 0.401 | **0.01**58 |
| PCRR | 0.0648 | **0.01**92 | 0.8893 | 0.0636 |
| PLICL | 0.1808 | 0.212 | 0.3812 | 0.1817 |
| PLICR | 0.3346 | 0.177 | 0.8341 | 0.3339 |
| PTRL | **0.00**92 | 7.00E-04 | 0.1108 | **0.01**13 |
| PTRR | **0.01**86 | **0.00**24 | 0.4502 | **0.01**99 |
| RLICL | 0.0744 | **0.00**63 | 0.6988 | 0.0793 |
| RLICR | 0.0863 | **0.04**53 | 0.5162 | 0.0806 |
| SCC | 0.0693 | 0.5026 | **0.01**53 | 0.0707 |
| SCRL | 0.0512 | 0.0544 | 0.8116 | **0.04**72 |
| SCRR | **0.04**01 | 0.0519 | 0.9694 | **0.04**05 |
| SFOL | **0.01**41 | 0.9303 | 0.1856 | **0.01**64 |
| SFOR | 0.2315 | 0.4269 | 0.9796 | 0.2418 |
| SLFL | 0.1068 | 0.1104 | 0.5579 | 0.1013 |
| SLFR | 0.1047 | 0.1685 | 0.2992 | 0.107 |
| SSL | **0.00**45 | **0.01**16 | 0.2068 | **0.00**64 |
| SSR | **0.02**95 | **0.01**92 | 0.449 | **0.02**43 |
| UNCL | 0.5061 | **0.01**28 | **0.00**13 | 0.5119 |
| UNCR | **0.03**18 | 0.5972 | **0.00**87 | **0.03**19 |
